# Reading, Writing, and English Course Pathways when Developmental Education is Optional: Course Enrollment and Success for Underprepared First-time-in-College Students

**DOI:** 10.1080/10668926.2017.1391144

**Published:** 2017-11-10

**Authors:** Chenoa S. Woods, Toby Park, Shouping Hu, Tamara Bertrand Jones

**Affiliations:** Center for Postsecondary Success, Florida State University, Tallahassee, FL, USA

## Abstract

Academic underpreparedness is an issue for many first-time-in-college students, particularly those entering community colleges. Whereas many underprepared students enroll in developmental education, research has indicated that traditional remediation may not increase students’ chances for success. Therefore, states and colleges have begun to implement new course placement strategies to increase the accuracy of initial course placement and new instructional approaches to better serve their developmental students. Specifically, in 2013, the state of Florida passed Senate Bill 1720 which redesigned developmental coursework and placement policies across the Florida College System. The reform lifted developmental education placement exam testing and course enrollment requirements for certain exempt students, irrespective of prior academic preparation or achievement. The current study focuses on these exempt students—those who had the option to bypass developmental education—who were also underprepared, and their initial course selection and subsequent success in their gateway (introductory college-level) English course. Using statewide student-level data and logistic regression techniques, the results indicated that level of preparation was related to students’ course enrollment and gateway English course success. Students slightly underprepared in reading or writing were more likely than severely underprepared students to enroll in the gateway English class, relative to a developmental reading or writing course. In reading and writing, slightly underprepared students were more likely to pass English, relative to severely underprepared students. The authors consider the findings in light of recent national changes to developmental education and offer recommendations for policy and practice.

Academic underpreparedness is an issue for many first-time-in-college (FTIC) students, particularly those entering community colleges. Approximately 60% of public community college students enroll in at least one developmental, or remedial, course (NCES, 2013). Developmental education (DE) courses have been the primary method in which colleges support students who enter college without college-level skills, but only 20% of students who enroll in DE complete the next college-level course within two years (Complete College America, 2016). Within the past decade, DE has come under scrutiny due in part to the large number of students it serves and the high costs—approximately $154 million per academic year in the state of Florida alone (Underhill, 2013). Further, research has indicated that the high costs of DE may not increase students’ chances for success (Bettinger & Long, 2009; Boatman & Long, 2010; Calcagno & Long, 2008; Lesik, 2006; Martorell & McFarlin, 2011; Moss & Yeaton, 2006; Scott-Clayton & Rodriguez, 2015).

Therefore, states and colleges have begun to implement new course placement strategies as well as new and innovated instructional approaches to increase the accuracy of initial course placement and to better serve their developmental students. For example, some states now afford students greater flexibility in terms of whether they are required to take DE. Instead of relying solely on a placement exam, placement strategies have included implementing a hierarchical placement system based on high school GPA and test scores, “boosts” into higher courses by considering high school transcript information in addition to test scores, and other ways of including multiple measures in the placement process (Kalamkarian, Raufman, & Edgecombe, 2015; Ngo & Kwon, 2015). At the same time, states as well as individual community colleges have begun exploring ways to change how DE is taught. For example, co-requisite courses combine enrollment in a college-level course with additional support often in the form of a developmental companion course or required tutoring or lab hours. Other instructional strategies colleges have implemented include modularized courses in which students complete only the modules in which they are deficient, compressed courses that shorten a semester-long course into eight- or twelve-week sessions, and contextualized courses that teach basic skills combined with content within majors or major-course pathways (Gardenhire, Diamond, Headlam, & Weiss, 2016; Kalamkarian et al., 2015; Perin, 2011).

In 2013, the state of Florida passed Senate Bill 1720 (SB 1720), which redesigned developmental coursework and placement policies across the Florida College System (FCS; formerly the community colleges). Florida’s DE reform lifted DE placement exam testing and course requirements for certain *exempt* students, irrespective of prior academic preparation or achievement. Thus, as of fall 2014, underprepared students are allowed to enroll in college-level courses in reading, writing, and math, as well as other introductory-level courses across disciplines.

In this study, we focused on these exempt students—those who have the option to bypass DE—and their initial course selection and subsequent success in the gateway (introductory college-level) English course. In Florida, both DE reading and DE writing are required before taking English Composition 1 for non-exempt students, depending on their placement test scores. Although placement testing is now optional for exempt students, many have scores from taking the test in high school, giving us the unique opportunity to analyze the enrollment patterns and course passing rates for students who would have previously been required to take DE but now have the option to bypass it.

We contribute to the existing body of research on DE in several unique ways. Many previous studies examined what would happen to students who were deemed just underprepared if they were allowed access into the gateway course instead. The current study models enrollment and subsequent success for underprepared students who had the option to enroll directly into gateway English courses. Further, we estimate how the degree of students’ underpreparedness is related to passing the first college credit-bearing course in English. This expands on what previous studies have explored in that they often focus on students who only marginally missed the placement exam cut score. Lastly, since DE reform is becoming more widespread across the country, this study will shed light on course enrollment and completion patterns when DE becomes optional and will have implications for practitioners and policymakers in other contexts.

## Literature review

Informing our analyses are three related bodies of literature. First, we review a series of existing studies that have documented the student characteristics and specific kinds of precollege academic preparation associated with success in college-level courses and that will serve as important control variables in our analytic models. Second, we discuss the major studies that have examined DE effectiveness, including placement policy. Third, we discuss recent DE reform measures adopted by various states, including a brief overview of four different DE instructional strategies that have been implemented in a number of locations, including Florida. We conclude by providing greater detail on the recent DE policy reform in Florida.

### College readiness

Much of the relevant college readiness literature has focused on malleable contributions to success, or factors that students, schools, programs, and colleges can change. Many of these factors are related to students’ high school experiences and transcript data, notably, coursework and performance. High school grades, often measured as an overall GPA, have been associated with college readiness and student success (Belfield & Crosta, 2012; Bridgeman, Pollack, & Burton, 2008; Hoffman & Lowitzki, 2005; Westrick, Le, Ribbins, Radunzel, & Schmidt, 2015). For example, high school GPA has had moderate to strong correlations with measures of first-year and second-year college GPA and second- and third-year retention (Westrick et al., 2015). Further, high school GPA alone can predict college-level English and math course success (Scott-Clayton, 2012).

High school courses and course rigor have also been shown to have strong relationships with college outcomes (Adelman, 2006; Jonas et al., 2012). For example, advanced levels of high school courses in English, math, science, and languages other than English (i.e., foreign language) have been associated with higher odds of success in gateway courses (Woods, Park, Hu, & Bertrand Jones, 2017). Further, high school Advanced Placement (AP) course and exam participation have been identified as positive predictors of college preparation, choice, admissions, and early success (Chajewski, Mattern, & Shaw, 2011; Scott, Tolson, & Lee, 2010). Although some research has demonstrated unclear relationships between participating in AP courses and students’ college success (Klopfenstein & Thomas, 2009; Thompson & Rust, 2007), others have found that AP exam scores are associated with first-year college GPA (Scott et al., 2010; Shaw, Marini, & Mattern, 2012). For example, students who earned scores of three or higher on their AP English language and AP Literature exams have been shown to have higher average college GPAs in their first semester than students who earned lower scores (Scott et al., 2010). AP course participation has also been associated with a higher likelihood of attending four-year institutions (Chajewski et al., 2011; Finkelstein & Fong, 2008). Further, Stephan, Davis, Lindsay, and Miller (2015) found that in Indiana, community college students who took dual-credit courses or AP courses had higher rates of early college success as measured by earning all attempted credits and persisting to the second year. These students were also less likely to enroll in remedial coursework. The same study found that students who earned a Core 40 diploma, which includes new course and credit requirements set by the state of Indiana, or a Core 40 diploma with honors were more likely to be successful on these same measures. The authors note, however, the inability to make causal claims from their study.

Interventions and programs implemented before the student matriculates into college have been shown to be effective methods of increasing students’ college readiness. Boot camps, bridge programs, and remediation while in high school are just some of the ways high schools and colleges intentionally implement programs to improve college readiness. Boot camps and comparable programs are designed to decrease the number of students assigned to DE prior to enrollment and do so by providing short but intense interventions (Sherer & Grunlow, 2010). In Sherer and Gunlow’s (2010) assessment of 14 math boot camps and similar programs at 10 colleges in seven states, they found that the goals of the programs were to improve students’ math placement test scores in order to begin in a college-level math course; improve math understanding for success in later courses; develop study skills and other college-readiness skills; develop relationships with peers, faculty, and others; and/or inform students of college services such as financial aid and support services. However, effects for these types of programs may be mixed. For example, Cabrera, Miner, and Milem (2013) found that effects on GPA from a summer program were mitigated once controlling for first-year college experiences. Further, in an assessment of summer bridge programs at six community colleges and universities, only some of the colleges had significant differences between pretest and posttest scores on placement exams such as the ACCUPLACER and COMPASS (Kallison & Stader, 2012).

Remediation during high school can also affect students’ course placement and success. For example, the Early Assessment Program (EAP) in California provided high school juniors with information about their college readiness and provided the opportunity for remediation in high school, prior to college entry. Howell, Kurlaender, and Grodsky (2010) found that EAP participants had reduced rates of DE placement when attending a moderately competitive regional four-year institution in the California State University system. Florida previously had a similar program, in which high school students took the Postsecondary Education Readiness Test (PERT) and had the opportunity for remediation during high school. However, the program was generally phased out in 2014.

Researchers and practitioners alike focus on reading and writing readiness because of their importance in students’ later success; reading and writing skills are requisite in most other college-level courses. Academic literacy includes reading, writing, and speaking the academic language common in textbooks and other learning materials (Maloney, 2003). This language tends to be more difficult for students than more casual or fiction writing, as it has a high density of information-rich words, complex sentence structures, and an authoritative voice (Snow, 2010). Research has highlighted the purpose of content area literacy as the teaching of basic reading skills, such as scanning the table of contents and attending to headings and subheadings, which can be used in a variety of disciplines (Shanahan & Shanahan, 2012). One way to measure reading and writing skills early in college is through grades in the English composition course. Kovacs (2016) reported that earning an A or a B in the course was related to higher rates of college graduation, compared to earning a C or lower. Likewise, there is evidence that completing gateway English, particularly within the first year of college, is related to higher rates of earning a college credential (Leinbach & Jenkins, 2008). These findings indicate that reading and writing skills are important for longer-term measures of student success.

### Developmental education effectiveness and instructional strategies

Existing research on the effectiveness of DE has yielded inconsistent conclusions; results often indicate nonsignificant or negative outcomes. For example, some researchers have argued that there is little association between remediation and early college success (Martorell & McFarlin, 2011; Scott-Clayton & Rodriguez, 2015; Ulmer, Means, Cawthon, & Kristensen, 2016). Martorell and McFarlin (2011) employed a regression discontinuity research design and, using administrative data from Texas, found that there was little indication that remediation benefited student academic outcomes or longer-term economic outcomes. The authors noted nonsignificant relationships between community college students’ remediation and total credits earned, transfer status, degree attainment at four, five, or six years after entry, and income earned. Other research has shown that when assigned to math remediation, students had an equal likelihood of enrolling in college, earning a degree, transferring, persisting, or dropping out, and earned an equivalent number of college-level credits compared to students who were enrolled assigned to college-level math (Scott-Clayton & Rodriguez, 2015).

Worse yet, some research has declared significant negative relationships with DE assignment or participation and student outcomes. For example, Scott-Clayton and Rodriguez (2015) used a regression discontinuity design and concluded that students assigned to developmental reading (instead of college-level English) were about 16 percentage points less likely to take a college-level English course, 13 percentage points less likely to pass a college-level English course, and 5 percentage points less likely to earn an associate’s degree. Similarly, students enrolled in North Carolina’s community colleges who were required to take developmental English were 23.9 percentage points less likely to ever pass the college-level English course and 17.4 percentage points less likely to earn an associate degree or pass 10 or more transfer-level courses within 4 years of entry (Clotfelter, Ladd, Muschkin, & Vigdor, 2015).

Performance in DE courses, however, has been shown to be positively related to performance in the associated gateway course. Ulmer and colleagues (2016) found that including students’ DE English course grades explained 8–9% more variation in the models and were modestly, yet positively, predictive of grades in the introductory college-level English course. However, the methods used in this study cannot support a causal relationship between the grades in the two courses. Often, the problem with success in DE pathways is that students don’t reenroll in the following course to complete the sequence (Bailey, Jeong, & Cho, 2010).

Research has also explored the extent to which placement tests—the tool traditionally used to place students into DE or college-level courses—are effective at gauging academic ability and potential for success. Some researchers have concluded that standardized tests do not always lead to accurate course placement (Scott-Clayton, 2012; Scott-Clayton, Crosta, & Belfield, 2014). In fact, placement exams have been shown to result in “severe mis-assignment,” or the placement of students in inappropriate courses (Scott-Clayton et al., 2014, p. 371). Overplacement is when students test into a course that is too difficult and where they have a reduced likelihood of success. Underplacement, which is more common, occurs when a student is placed into a DE course when they were predicted to be successful in the gateway course; thus, these students are underplaced compared to their ability. Interestingly, modestly raising placement exam cut scores (to facilitate college-level course entry for more students) does not significantly affect placement accuracy (Ngo & Melguizo, 2016).

The lackluster findings regarding DE participation and success have resulted in course redesigns. Instructional modalities, including those approved under SB 1720 (modularized, contextualized, compressed, and co-requisite) have been implemented in colleges and states across the country. For example, Virginia and North Carolina integrated their DE reading and writing courses, which reduced the time needed to complete the remedial sequence (Kalamkarian et al., 2015, May). The new courses included co-requisite supports and contextualized reading and writing lessons that are related to the college-level English course.

Co-requisite courses have been associated with higher proportions of students completing their gateway courses within their first semester (Complete College America, 2016). The Accelerated Learning Program (ALP) at the Community College of Baltimore County is one example of a well-designed co-requisite course model. Students testing into the higher DE English levels were placed into college-level English courses and co-enrolled in an associated ALP course taught by the same instructor. Program participation was associated with higher rates of passing English 101 and 102 (Jenkins, Speroni, Belfield, Jaggars, & Edgecombe, 2010). Modularized courses, or those that focus students’ time and effort on modules in which they are deficient, have been associated with higher rates of passing the DE course (Okimoto & Heck, 2015). Contextualized instruction, or basic skills taught in the context of real-world problems and major course content, has been related to earning more college credits and an increased likelihood of persistence (Zeidenberg, Cho, & Jenkins, 2010). Compressed courses, or those aimed at accelerating DE by shortening the time spent in each course, have been shown to increase students’ likelihood of passing their college-level English course by 12 to 22 percentage points (Edgecombe, Jaggars, Xu, & Barragan, 2014).

### Developmental education reform measures

At the same time, different states and institutions have adopted a variety of methods to measure college readiness. For example, in Florida, between 2011 and 2014, every FCS student was required to take the Postsecondary Education Readiness Test (PERT), a standardized placement test to determine college readiness in reading, writing, and math. Other states and colleges have used commercial tests, such as the ACT, PSAT, SAT, ASSET, COMPASS, or ACCUPLACER, produced by companies such as ACT Inc. or the College Board (Clotfelter et al., 2015). However, there have been debates about whether these tests accurately reflect what is taught in high school or what is required from students once they reach college; there may be disagreement between different types of colleges, even within the same state, as to what determines college readiness (Burdman, 2015).

### The Florida context and recent DE reform

The FCS is composed of 28 colleges and 68 campuses. In the 2014–2015 academic year, the annual student headcount at these open-access institutions was more than 813,000 students, 35% of whom were enrolled full-time, and 58% of whom were racial/ethnic minority students (Florida College System, 2017). The colleges vary in size (from serving around 1,000 students to more than 66,000 students), the racial/ethnic backgrounds of their students (from 5% to 33% Black and 3% to 68% Hispanic), and the financial backgrounds of their students (36% to 74% receiving a Pell grant; National Center for Education Statistics, 2017). Students in FCS institutions are from rural, suburban, and urban settings.

In 2013, the Florida legislature passed SB 1720, which dramatically reshaped DE placement, requirements, and course offerings throughout the state’s community colleges. SB 1720 made DE optional for a large portion of students. *Exempt* students are those who entered a Florida public high school in 2003–2004 or later and subsequently graduated with a standard high school diploma, or active duty military personnel. Under the current legislation, these exempt students, many of whom are underprepared, may opt out of placement testing and enroll directly into a college-level gateway course, which places the onus of enrollment decisions largely on students.

SB 1720 made DE placement testing optional for exempt students, but under prior legislation of House Bill 1255, the PERT was administered to high school students in the 2011 to 2014 academic years (Florida College System, n.d.). More specifically, students who earned a score of 2 or 3 on the Florida Comprehensive Assessment Test in reading (on a scale of 1 through 5) were required to take the PERT in their junior year of high school. Although this legislation has since been phased out, because they tested in high school, many incoming college students have PERT scores despite being exempt based on earning a standard high school diploma or their military status.

As part of SB 1720, colleges were also required to offer DE courses in at least two of four new instructional modalities: modularized, compressed, contextualized, and co-requisite, and all incoming students were required to meet with an academic advisor. Thus, whereas only exempt students were excused from DE placement and testing, *all* students who enrolled in a DE course were taught with new instructional methods.

We may be able to learn some lessons from a study examining this DE reform in Florida for underprepared math students. Park, Woods, Tandberg, Hu, and Bertrand Jones (2016) found that many students who would have been placed into DE math under the old placement policies chose to enroll in Intermediate Algebra, the gateway math course, but some also enrolled in no math at all. However, the students who did enroll in DE math benefitted from doing so and were more likely to pass the gateway math course than their underprepared peers who did not take DE and the gateway course together. Further, more prepared students had higher odds of passing the Intermediate Algebra course.

The purpose of this study is to document the enrollment patterns of underprepared FTIC students—students who would have previously been required to take DE courses—now that DE is optional in Florida, and whether the students who opted to bypass DE reading and writing courses were successful in passing the gateway English course. More specifically, we asked the following research questions:

1. What reading, writing, and English courses do underprepared FTIC students choose to take now that DE is optional, and do these choices differ by the level of preparation?
2. How successful are underprepared FTIC students who bypass DE and instead enroll in gateway English courses, and does this differ by level of preparation?

## Methods

### Data and sample

After receiving approval from our Institutional Review Board, we received data for this study from Florida’s Education Data Warehouse (FL-DOE). We obtained statewide administrative data of FTIC students entering the FCS in 2009–2010 to 2014–2015 fall cohorts. For the purpose of this study, we limited our sample to students who began college in 2014, the first year the DE reform was fully enacted. We also limited our sample to students who had complete high school records and PERT scores in both reading and writing. For ease of interpretation, we eliminated students who co-enrolled in a developmental and a gateway course within the same semester. Although this enrollment pathway is worthy of future study, modeling outcomes for such a small group of students spread across multiple campuses throughout the FCS would not yield useful practical results. Our final sample consisted of 16,796 students, 25% of the incoming FCS FTIC cohort in 2014.

In the overall sample, more than 37% of students were White, 35.2% of students were Hispanic, 21.8% were Black, and 5.4% were of another race/ethnicity (see Table 1). Slightly more than half were women (51.6%) and were eligible for free- or reduced-price lunch in high school (54.2%). In terms of high school preparation, the majority of students had completed Algebra 2 (72.7%), but fewer earned credits in trigonometry (4.0%) or another advanced math course (14.9%). Nearly 45% had honors English credit, and 8.2% had earned Advanced Placement (AP) credit in an English course.

**Table 1.** Sample characteristics, by level of reading preparation.

		Severely underprepared	Moderately underprepared	Slightly underprepared	College-ready
	Overall sample	PERT ≤83	PERT = 84–94	PERT = 95–105	PERT ≥106
Student Background Characteristics (S)					
White	37.6%	26.5%	28.7%	36.4%	45.8%
Black	21.8%	36.7%	30.6%	23.0%	12.9%
Hispanic	35.2%	31.8%	35.0%	35.4%	35.9%
Other Race/Ethnicity	5.4%	4.9%	5.6%	5.2%	5.4%
Women	51.6%	48.3%	55.5%	55.4%	47.2%
Free/Reduced Lunch	54.2%	62.6%	61.1%	55.7%	47.4%
High School Academic Preparation (HS)					
Algebra 2	72.7%	59.2%	69.3%	75.8%	75.2%
Trigonometry	4.0%	2.2%	3.5%	4.6%	4.2%
Other Advanced Math	14.8%	6.0%	9.9%	14.9%	19.4%
Honors English	44.8%	16.8%	31.4%	46.0%	57.4%
AP English	8.2%	1.5%	3.8%	7.6%	12.5%
N	16,796	9.3%	19.9%	31.9%	39.0%

### Analytical approach

To investigate reading, writing, and English course enrollment patterns, we adapted an analytic approach from Park, Woods, Tandberg et al. (2016) who analyzed math course enrollment patterns for underprepared students following DE reform in Florida. Specifically, in the current study, there are two outcome variables of interest: 1) enrollment in developmental reading, developmental writing, and the gateway English course and 2) passing the gateway course. The gateway English course, English Composition 1 (ENC 1101) is the traditional college-level course in both of the reading/English and writing/English pathways. We created two separate enrollment variables to capture enrollment in the reading/English and writing/English pathways. That is, we coded enrollment in no course as 0, enrollment in a developmental course as 1, and enrollment in the gateway course as 2. The second dependent variable is passing the gateway course; students were coded as passing if they had earned a C- or higher.

The key independent variables were students’ level of preparedness defined by PERT scores in reading and writing. Since the PERT reading and writing scores were separate, it was possible that a student was placed into different levels of preparation based on the test subject. Students in the *severely* underprepared reading group had PERT scores of 83 or lower (out of 150), which placed them into the lowest level of DE. Traditionally, based on PERT cut scores, *moderately* underprepared students and *slightly* underprepared students were placed into the higher DE course; these PERT scores ranged from 84 to 105. To determine students who were still underprepared but were close to being college ready, we split this group. Students who were *moderately* underprepared had reading PERT scores ranging from 84 to 94, and students who were *slightly* underprepared had reading PERT scores between 95 and 105. In doing so, about 62% of students who would have traditionally been placed into the higher DE reading course were determined s*lightly* underprepared. *College-ready* students had reading scores of 106 or higher.

More than a third of students in the overall sample were college ready in reading (39.0%), and another third were slightly underprepared (31.9%); 19.9% were moderately underprepared, and 9.3% of students were severely underprepared (Table 1). Students of different background characteristics and high school preparation were distributed differentially among the levels of preparation by PERT score. For example, whereas White students made up 45.8% of the college-ready sample in reading, Black students made up just 12.9% of students with the highest levels of preparation. Conversely, Black students composed more than a third of the severely underprepared group (36.7%), whereas 26.5% of these students were White. Interestingly, equal proportions of Hispanic students were represented among each preparation level. Low-income students were overrepresented in the lower achievement levels. As might be expected, most students with advanced courses in high school were college-ready in reading. That is, just 59.2% of the least prepared students had completed Algebra 2, compared with 75.2% of college-ready students. We found similar patterns of different magnitudes for other high school courses.

We constructed similar PERT score categories for writing students. *Severely* underprepared students scored 89 or lower (out of 150) in writing. Traditionally, based on PERT writing cut scores, *moderately* underprepared students and *slightly* underprepared students were placed into the higher DE course, with scores ranging from 90 to 102. Again, we split this group into *moderately* and *slightly* underprepared students. Students who were *moderately* underprepared in writing had PERT scores ranging from 90 to 96, and students who were *slightly* underprepared had PERT scores between 97 and 102. Thus, about 56% of students who would have traditionally been placed into the higher DE writing course were categorized as *slightly* underprepared. *College-ready* students had writing scores of 103 or higher.

In the overall writing sample, 46.3% of students were college-ready, another 21.0% were slightly underprepared, 16.6% were moderately underprepared, and 17.1% of students were severely underprepared (Table 2). We found many of the differences that were present in the reading sample present in the writing sample as well. Again, equal proportions of Hispanic students were present among each preparation level, and low-income students were overrepresented in the lower achievement levels. Similar patterns of high school preparation also emerged.

**Table 2.** Sample characteristics, by level of writing preparation.

		Severely underprepared	Moderately underprepared	Slightly underprepared	College-ready
	Overall sample	PERT ≤89	PERT = 90–96	PERT = 97–102	PERT ≥103
Student Background Characteristics (S)					
White	37.6%	20.6%	31.5%	37.6%	46.3%
Black	21.8%	39.2%	27.1%	21.4%	13.5%
Hispanic	35.2%	35.2%	35.9%	36.3%	34.4%
Other Race	5.4%	5.1%	5.5%	4.6%	5.8%
Women	51.6%	50.3%	55.0%	54.4%	49.5%
Free/Reduced Lunch	54.2%	68.0%	59.2%	53.7%	47.4%
High School Academic Preparation (HS)					
Algebra 2	72.7%	64.3%	70.5%	75.9%	75.2%
Trigonometry	4.0%	2.2%	3.7%	4.6%	4.6%
Other Advanced Math	14.8%	7.7%	11.2%	14.9%	18.8%
Honors English	44.8%	20.0%	35.7%	46.2%	57.0%
AP English	8.2%	2.1%	4.0%	7.0%	12.5%
N	16,796	17.1%	16.6%	21.0%	45.3%

First, we conducted a series of single-factor ordered logistic regression models to determine how background characteristics and high school coursework predicted students’ PERT score categories. These findings are presented as odds ratios where values greater than 1 are associated with being classified into higher levels of preparation. Next, we analyzed descriptive statistics of students’ enrollment patterns disaggregated by level of preparation. Then, to uncover the relationships between levels of preparation and reading and writing enrollment patterns for underprepared students net of background characteristics and high school preparation, we conducted two standard multinomial logistic regression equations modeling reading and writing separately:

$$\mathrm{Pr}\left(Y_{i}=j\right)=\;\frac{exp\left(\beta_{j0}+\beta_{j1}moderately_{i}+\beta_{j2}slightly_{i}+\delta_{j}S_{i}+\gamma_{j4}HS_{i}\right)}{\sum_{k=0}^{K}exp\left(\beta_{j0}+\beta_{j1}moderately_{i}+\beta_{j2}slightly_{i}+\delta_{j}S_{i}+\gamma_{j4}HS_{i}\right)}$$

Under this specification, *Y_i_* is the reading or writing enrollment outcome *j* for individual *i*: enrollment in no English course pathway, enrollment in a developmental course, or enrollment in the gateway English course. *Moderately* and *slightly* are dichotomous indictors for level of preparation (*severely* is the reference group), and *S* and *HS* are vectors that encompass student background variables and high school course-taking indicators. Student characteristics included race/ethnicity, gender, and free- or reduced-lunch eligibility, a crude indicator of low-income status. More specifically, we included indicators for students of Black, Hispanic, and “other” race/ethnicity (including Asian Americans, Pacific Islanders, multiracial students, and students of an unknown race) in the model and used White as the reference category; men were the reference category for gender. The high school variables were indicators for whether students had earned high school credit for Algebra 2, Trigonometry, another advanced math course (including pre-calculus, calculus, and statistics), English Honors, and Advanced Placement Honors. Each of these indicators were coded as 1 if the student had earned credit in the course, and 0 otherwise. We include these indicators of high school coursework as a way of measuring prior academic preparation and experience with higher-level course material.

We present our results as relative risk ratios. This model allows us to determine whether FTIC students who were *moderately* and *slightly* underprepared enrolled in the three enrollment pathways at different rates compared to FTIC students in the *severely* underprepared category, after we controlled for the aforementioned background factors. Then we presented predicted probabilities of enrollment in the various reading/English and writing/English pathways separated by level of preparation (all other variables set to the within-group mean), with taking DE reading or writing as the base category. We also present the 95% confidence intervals for the predicted probabilities. This allows us to pinpoint significant differences across preparation levels if, for instance, the predicted probability for *moderately* underprepared falls outside the confidence interval for *slightly* underprepared.

To understand underprepared FTIC students’ success in their gateway course, and whether these relationships varied by level of preparation, we implemented the following standard logistic regression equation separately for reading and writing pathways:

$$Logit\left(Y_{i}\right)=\beta_{0}+\;\beta_{1}{\left(moderately\right)}_{i}+\;\beta_{2}{\left(slightly\right)}_{i}+\;\delta {\left(S\right)}_{i}+\;\gamma {\left(HS\right)}_{i}$$

Under this specification, *Y_i_* is a dichotomous indicator of whether student *i* passed the gateway English course with a grade of C- or better and *moderately* and *slightly, S*, and *HS* are as before. Therefore, we compared the estimates of *moderately* and *slightly* to *severely* underprepared students, which allowed us to examine whether FTIC students who had different levels of preparation were successful in English Composition 1. Again, we presented these results with predicted probabilities and the associated 95% confidence intervals.

## Results

In the following sections, we present our findings. We begin with the results from our ordered logistic regression equations of all students in our sample, which help us determine which students are prepared at which levels in reading and writing. Then, we present descriptive statistics of students’ English enrollment pathways only for students underprepared in reading. Next, we discuss descriptive statistics of enrollment patterns, the findings from the multinomial logistic regression, and the associated predicted probabilities and confidence intervals for the underprepared reading sample. We repeat this pattern of exploring the results of each analysis for underprepared writing students, beginning with a review of the descriptive statistics. We conclude the results section with our findings of underprepared students’ likelihood of passing the gateway English course, first for the reading sample, then for the writing sample.

### Reading and writing samples

In the overall reading sample, results from the ordered logistic regression models determined that Hispanic students and women were more likely to be placed into the next higher category of PERT scores, but Black students and students who were eligible for free- or reduced-price lunch were more likely to be placed into the next lower category (Table 3). Interestingly, all of the high school preparation factors were significantly predictive of being placed in the higher PERT category.

**Table 3.** Results from ordered logistic regression analyses.

	Reading sample			Writing sample		
	O.R.	S.E.	*p*	O.R.	S.E.	*p*
Student Background Characteristics (S)						
White	1.456	0.060	0.000	1.843	0.078	0.000
Black	0.613	0.026	0.000	0.516	0.022	0.000
Hispanic	1.085	0.043	0.039	1.039	0.042	0.344
Other Race	0.990	0.083	0.907	0.917	0.080	0.318
Women	1.140	0.043	0.001	1.127	0.043	0.002
Free/Reduced Lunch	0.793	0.031	0.000	0.637	0.025	0.000
High School Academic Preparation (HS)						
Algebra 2	1.683	0.070	0.000	1.530	0.065	0.000
Trigonometry	1.573	0.160	0.000	1.662	0.174	0.000
Other Advanced Math	1.917	0.119	0.000	1.738	0.107	0.000
Honors English	2.474	0.102	0.000	2.439	0.101	0.000
AP English	2.684	0.259	0.000	2.455	0.240	0.000

In the overall writing sample, White students and women had higher odds of being placed into the next highest PERT score grouping, and Black students and students eligible for free- or reduced-price lunch were also more likely to be in the next lower category. In terms of high school preparation, students who had earned credit in Algebra 2, Trigonometry, another advanced math course, Honors English, or AP English had higher odds of being placed into the highest PERT score category. Overall, these results indicated that student characteristics and high school background factors were related to students’ PERT score grouping; most notable was that White and Black students were disproportionately represented in college-ready and severely underprepared groups, respectively. These results reinforced the descriptive statistics detailed in Tables 1 and 2.

It is worth noting that the ordered logistic regression analyses included students who were college ready. We retained college-ready students in the sample as a means of understanding the distributions of students’ background characteristics across all PERT score categories. The following analyses focus solely on underprepared students.

### English Enrollment pathways

#### Reading

**Figure 1.**
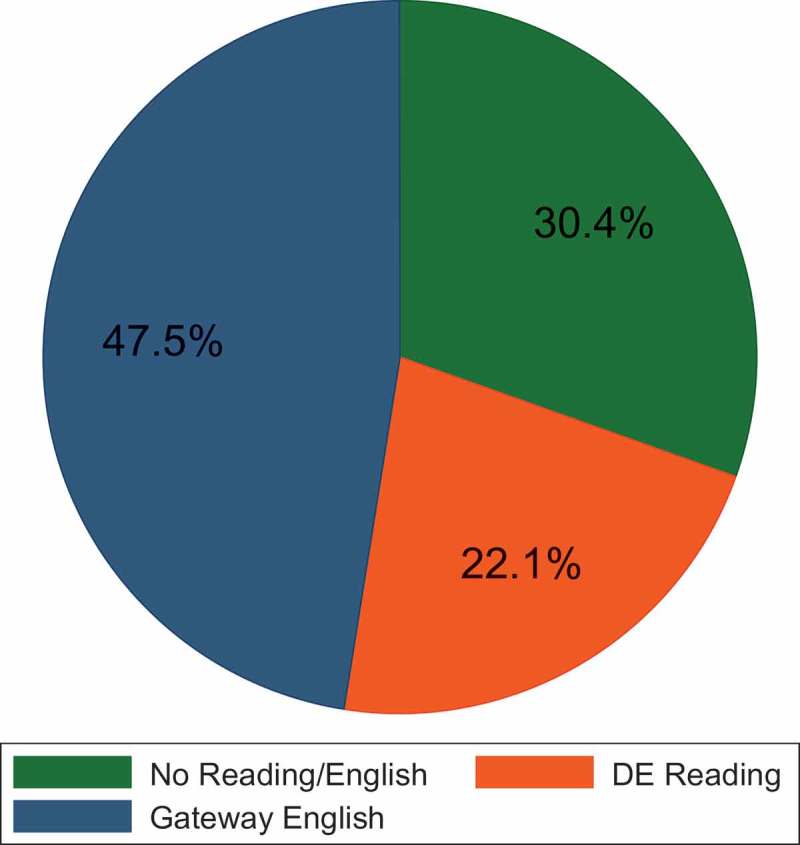
Reading/English enrollment pathways.

When we focused on the reading/English enrollment patterns of underprepared students, nearly half (47.5%) enrolled directly into the gateway course, and the remaining students enrolled in a developmental reading class (22.1%) or bypassed a reading/English pathway altogether (30.4%; Figure 1). Table 4 displays enrollment patters disaggregated by level of preparation. More severely underprepared students choose no reading/English pathway (39.7%) than a DE reading course (29.0%) or ENC 1101 (31.3%). However, more moderately (41.6%) and slightly (55.9%) underprepared students chose the gateway course over other enrollment options.

**Table 4.** Underprepared students’ enrollment patterns by level of preparation.

	No Reading		DE Reading		Gateway English		
	Number	Percent	Number	Percent	Number	Percent	Total
Severely Underprepared	620	39.7	453	29.0	488	31.3	1,561
Moderately Underprepared	1,087	32.6	862	25.8	1,389	41.6	3,338
Slightly Underprepared	1,412	26.4	945	17.7	2,992	55.9	5,349
Total	3,119	30.4	2,260	22.1	4,869	47.5	10,248
	No Writing		DE Writing		Gateway English		
	Number	Percent	Number	Percent	Number	Percent	Total
Severely Underprepared	911	31.7	1,042	36.2	922	32.1	2,875
Moderately Underprepared	768	27.6	757	27.2	1,256	45.2	2,781
Slightly Underprepared	914	25.9	619	17.5	1,997	56.6	3,530
Total	2,593	28.2	2,418	26.3	4,175	45.5	9,186

Multinomial logistic regression results revealed that level of preparation was significantly related to enrolling in ENC 1101. That is, in comparison to students who were severely underprepared, students who were slightly underprepared (RR = 1.914, *p *< .001) had higher odds of enrolling in the gateway course instead of a developmental course (Table 5). Further, level of preparation was also related to bypassing a reading/English pathway; in contrast to severely underprepared students, students who were moderately underprepared were less likely (RR = 0.878, *p *< .05) to bypass a reading/English pathway when compared to enrolling in a DE reading course. Although women were more likely to enroll in the gateway English course than a developmental course, students of color, as well as those eligible for free/reduced lunch were less likely to enroll in this course, as compared to a developmental reading course. Many of the same students also had lower odds of bypassing the reading/English pathway, meaning that of the three enrollment options, underrepresented minority and low-income students were most likely to enroll in the developmental reading course. As might be expected, students with higher levels of advanced high school coursework had higher odds of enrolling the gateway course than the developmental course. Interestingly, advanced English coursework was also related to not taking any reading/English course. A possible explanation for this is that these students have already completed the basic composition requirement through earning Advanced Placement credit.

**Table 5.** Multinomial logistic regression relative risk ratios for reading and writing enrollment patterns of underprepared students.

	Reading sample		Writing sample	
	No reading	Gateway English	No writing	Gateway English
Level of Preparedness				
Moderately Underprepared	0.878*	1.197	1.084	1.496***
	[0.057]	[0.112]	[0.068]	[0.112]
Slightly Underprepared	0.977	1.914***	1.513***	2.532***
	[0.078]	[0.163]	[0.089]	[0.166]
Student Background Characteristics (S)				
Black	0.741***	0.487***	0.691***	0.527***
	[0.051]	[0.037]	[0.047]	[0.041]
Hispanic	0.792***	0.649***	0.734***	0.690***
	[0.047]	[0.045]	[0.049]	[0.045]
Other Race	0.949	0.722*	0.849	0.787*
	[0.143]	[0.115]	[0.099]	[0.095]
Women	1.058	1.157*	0.937	1.068
	[0.060]	[0.069]	[0.055]	[0.054]
Free/Reduced Lunch	0.825***	0.829***	0.937	0.907
	[0.045]	[0.038]	[0.060]	[0.059]
High School Academic Preparation (HS)				
Algebra 2	1.075	2.033***	1.098	2.098***
	[0.064]	[0.119]	[0.070]	[0.119]
Trigonometry	0.967	1.203	0.900	1.075
	[0.140]	[0.168]	[0.187]	[0.191]
Other Advanced Math	1.137	1.483***	1.106	1.470***
	[0.124]	[0.122]	[0.113]	[0.132]
Honors English	1.192*	2.222***	1.188*	2.137***
	[0.086]	[0.144]	[0.093]	[0.131]
AP English	1.524*	1.712**	1.224	1.365
	[0.273]	[0.299]	[0.203]	[0.222]
Constant	1.719***	0.924	1.132	0.672***
	[0.165]	[0.100]	[0.091]	[0.052]
ll	−10100.00		−9215.084	
*x*^2^	4025.746		12537.104	
N	10,248		9,186	

*Note*.* *p *< 0.05, ** *p *< 0.01, *** *p *< 0.001

We present predicted probabilities with their associated confidence intervals to help us interpret our findings. Notably, of slightly underprepared students, or those closest to the PERT cut-point, just 19.5% enrolled in a DE reading course, a rate that was significantly lower than students with lower levels of preparation (Table 6). However, severely and moderately prepared students enrolled in DE reading at similar rates, as indicated by their overlapping confidence intervals. Slightly underprepared students enrolled in ENC 1101 at rates significantly higher than their moderately and severely underprepared peers (52.7% compared to 43.5% and 37.8%, respectively). Further, when examining the predicted probabilities vertically (for instance, the predicted probability or enrolling in ENC 1101, we note that none of the intervals overlap, suggesting that students at each level of preparation were predicted to enroll in ENC 1101 at significantly different rates. Interestingly, severely underprepared students chose to not enroll in any reading or English course at significantly higher rates (37.1%) than moderately underprepared (31.0%) or slightly underprepared (27.8%) students.

**Table 6.** Predicted probabilities of reading and writing enrollment.

	No reading (%)			DE reading (%)			Gateway English (%)		
	Low	Est.	High	Low	Est.	High	Low	Est.	High
Severely Underprepared	34.1	37.1	40.2	23.0	25.0	27.0	35.0	37.8	40.6
Moderately Underprepared	30.5	31.9	33.3	23.2	24.6	26.0	42.0	43.5	45.0
Slightly Underprepared	26.7	27.8	28.9	18.5	19.5	20.4	51.1	52.7	54.3
	No Writing			DE Writing			Gateway English		
	Low	Est.	High	Low	Est.	High	Low	Est.	High
Severely Underprepared	29.0	30.5	32.1	30.8	32.6	34.4	35.1	36.9	38.6
Moderately Underprepared	25.9	27.6	29.4	25.9	27.4	29.0	42.8	44.9	47.0
Slightly Underprepared	26.1	27.5	28.9	18.7	19.7	20.7	51.1	52.8	54.5

#### Writing

**Figure 2.**
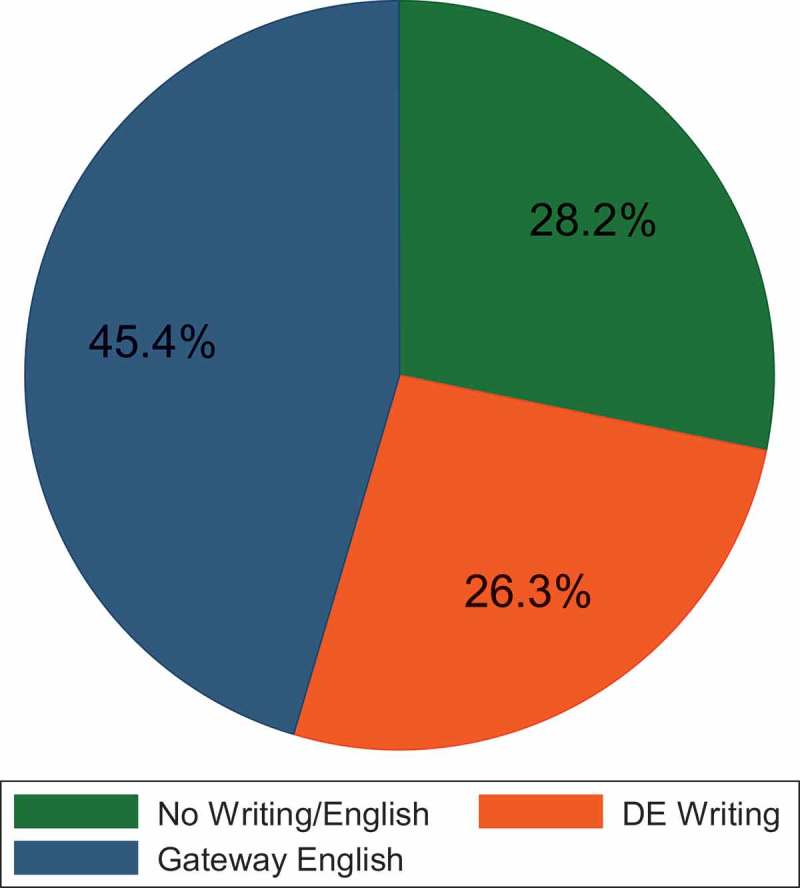
Writing/English enrollment pathways.

Descriptively, similar patterns emerged in the writing enrollment pathways. About 45% of underprepared students enrolled in the gateway English course, 26.3% enrolled in a DE writing course, and 28.2% enrolled in no English or writing course (Figure 2). When we disaggregated writing enrollment options by level of preparation, however, severely underprepared students chose to enroll in no writing/English course (31.7%) at similar rates as the gateway course (32.1%; Table 4). Moderately underprepared and slightly underprepared students enrolled in ENC 1101 at higher rates than either of the other enrollment options (45.2% and 56.6%, respectively).

As with reading, levels of preparation were predictive of writing/English pathways for underprepared students. That is, relative to severely underprepared students, students who were slightly (RR = 2.532, *p *< .001) and moderately prepared (RR = 1.496, *p *< .001) were more likely to enroll in the gateway English course as compared to enrolling in a DE writing course (Table 5). Slightly underprepared students were also more likely than severely underprepared students to not enroll in DE writing or ENC 1101 (RR = 1.513, *p *< .001). Similar to the outcomes for reading enrollment, racial/ethnic minority students were less likely than White students to enroll in the gateway course or to bypass or delay the writing/English sequence, indicating higher odds of enrolling in a DE writing course, relative to their White peers. Again, students with more advanced high school coursework were more likely to enroll in the gateway course; however, only credit in Honors English was predictive of enrollment in the no writing/English course option.

Predicted probabilities indicated that slightly underprepared students enrolled in the gateway course at significantly higher rates than their less prepared peers (52.8% compared to 36.9% and 44.9%), and chose the DE writing course at the lowest rates (19.7% compared to 32.6% and 27.4%; Table 6). However, 27.5% of slightly underprepared students opted to enroll in no writing/English course at all, a rate significantly lower than the rate of severely underprepared students (30.5%). Overlapping confidence intervals indicated that moderately and slightly underprepared students bypassed a writing/English course at similar rates (27.6% and 27.5%, respectively).

### Gateway English success

#### Reading

As might be expected, students with higher levels of preparation were more likely to pass their gateway course. That is, students who were moderately (OR = 1.366, *p *< .01) and slightly (OR = 1.411, *p *< .001) underprepared in reading had higher odds of passing the course compared to their severely underprepared peers (Table 7). Black students had lower odds of successfully completing the course than White students, but women were more likely to pass than men. Predictably, students with more advanced high school preparation were also more likely to pass the course. Chi-squared tests indicated that slightly underprepared students and moderately underprepared students had similar odds of passing the course (*χ* ^2^ = .21, *p *> 0.05).

**Table 7.** Gateway completion for underprepared students.

Levels of preparedness	Reading	Writing
Moderately Underprepared	1.366**	1.322**
	[0.151]	[0.123]
Slightly Underprepared	1.411***	1.305**
	[0.146]	[0.114]
Student Background Characteristics (S)		
Black	0.687***	0.702***
	[0.059]	[0.066]
Hispanic	1.101	1.104
	[0.087]	[0.094]
Other Race	0.928	0.979
	[0.135]	[0.156]
Women	1.509***	1.435***
	[0.095]	[0.097]
Free/Reduced Lunch	0.886	0.901
	[0.060]	[0.066]
High School Academic Preparation (HS)		
Algebra 2	1.616***	1.628***
	[0.129]	[0.139]
Trigonometry	1.073	1.077
	[0.157]	[0.177]
Other Advanced Math	1.576***	1.483***
	[0.151]	[0.152]
Honors English	1.117	1.188*
	[0.075]	[0.086]
AP English	1.359*	1.368*
	[0.182]	[0.211]
Constant	0.799	0.844
	[0.097]	[0.096]
ll	−2979.094	−2579.36
*x*^2^	227.635	187.935
N	4,869	4,175

*Note*.* *p *< 0.05, ** *p *< 0.01, *** *p *< 0.001

Predicted probabilities indicated that 67.8% of students who were slightly underprepared in reading passed ENC 1101, but that was not statistically higher than the pass rate for students who were moderately prepared (67.2%; Table 8). However, just 60.3% of severely underprepared students passed the course, a rate significantly lower than students in either of the higher prepared groups, as indicated by the non-overlapping confidence intervals; moderately and slightly underprepared students were predicted to pass at statistically similar rates.

**Table 8.** Predicted probability of passing gateway English, by preparation level.

	Pass Gateway English (%)		
	Low	Est.	High
Reading			
Severely Underprepared	56.0	60.3	64.5
Moderately Underprepared	64.7	67.2	69.6
Slightly Underprepared	66.2	67.8	69.5
Writing			
Severely Underprepared	58.4	61.5	64.6
Moderately Underprepared	65.1	67.6	70.2
Slightly Underprepared	65.3	67.4	69.4

#### Writing

Students who were slightly (OR = 1.305, *p *< .01) and moderately (OR = 1.322, *p *< .01) underprepared in writing had higher odds of passing their English Composition 1 course as compared to students who were severely underprepared. (Table 7). Again, Black students had lower odds of passing the course than White students, but women were more likely to pass, as were students with more advanced high school coursework. Chi-squared tests indicated that students who were slightly and moderately underprepared in writing had similar odds of passing the course (*χ* ^2^ = 0.0, *p *> 0.05).

Predicted probabilities indicated that moderately (67.6%) and slightly (67.4%) underprepared students passed their English course at statistically similar rates, but severely underprepared students passed at much lower rates (61.5%; Table 8). Because the confidence intervals did not overlap, we determined that students in the severely underprepared group were predicted to pass the gateway English course at statistically significantly lower rates than students who were moderately or slightly underprepared. However, the confidence intervals for moderately and slightly underprepared students overlapped, indicating statistically similar predicted probabilities of passing the course.

## Discussion

This paper sought to document the enrollment rates of underprepared students in DE reading, DE writing, and gateway English courses, and passing rates for those underprepared students who enrolled directly in gateway English, after controlling for a number of student characteristics and precollege academic preparation. We were interested in students’ reading/English and writing/English course enrollment choices, the extent to which being underprepared was related to the course in which they enrolled, and the extent to which their preparation was related to passing the gateway course. Results revealed that given the option, many underprepared students enrolled directly into English Composition 1, but many also chose not to enroll in a reading, writing, or English course. Patterns were generally similar for reading and writing, in that slightly underprepared students were most likely to enroll in the gateway course and severely underprepared students were most likely to opt out of a reading/English or writing/English pathway during their first year. Finally, we found that moderately and slightly underprepared students passed gateway English at significantly higher rates than severely underprepared students.

It is not surprising that 46% to 48% of underprepared students chose to enroll in the gateway English course now that they have this option. Previous research has documented that under the recent Florida DE reform, students made enrollment choices based in large part on cost and time to degree (Park, Woods, Richard et al., 2016). It is possible that the students in the current study, particularly those who were closest to the college-ready cut point, opted to bypass DE and enroll in the gateway course for these same reasons. In addition, 28% to 30% of students choose to delay enrollment in any reading, writing, or English course; many of these students were severely underprepared. While these rates of delay may appear alarming, research on completing math course requirements shows that although initial enrollment is important, later enrollment may be just as important. That is, Wang, Wang, Wickersham, Sun, and Chan (2017) found that completing core math course requirements in the first, fourth, or fifth semesters was associated with an increased likelihood of earning a credential. Although the study did not include English requirements, it is possible that delaying the completion of core courses (i.e., not enrolling in ENC 1101 within the first year) may not have as detrimental effects as previously assumed.

Interestingly, in reading and in writing, both groups of higher prepared students had higher odds of passing the gateway course, relative to the least prepared students. This finding aligns with those of a similar study examining success in gateway math following Florida’s DE redesign (Park, Woods, Tandberg et al., 2016). Further, there wasn’t much variation in predicted probabilities of passing ENC 1101 across reading and writing. That is, students who were severely underprepared in reading were predicted to pass at 60.3%, whereas students severely underprepared in writing were predicted to pass at 61.5%. Similarly, across the moderately and slightly underprepared students in reading and writing, predicted probabilities of passing were 67.2% to 67.8%. This finding indicates that students who were underprepared in reading have similar odds of passing ENC 1101 as those who were underprepared in writing.

### Recommendations for policy and practice

We consider our findings in light of the national attention to DE reform and offer several recommendations for policy and practice. Given that students’ high school coursework was predictive of gateway course success, even after controlling for preparation level by PERT score, we argue, as others have, that a multiple measures placement policy may be the most appropriate way to place students into developmental or gateway courses (Melguizo, Kosiewicz, Prather, Bos, 2014; Ngo & Kwon, 2015; Scott-Clayton et al., 2014; Willet et al., 2015). High school coursework, perhaps in addition to test scores (when available), may be the best placement mechanism. Indeed, advanced high school coursework has been shown to predict success in gateway courses in Florida following this reform (Woods et al., 2017).

That said, and given that the levels of preparation were shown to be significant predictors of course completion, our study demonstrates that the PERT provides some value for advisors to guide students when engaging in the course registration process. However, because the test is now optional, fewer students will have scores that can help them understand their preparedness for college-level work. Thus, colleges may be wise to devise strategies that encourage exempt students to take the exam, even though it is optional. One way to incentivize optional test taking is to remove financial barriers such as exam fees and ease the testing process by having flexible testing hours and study guides. Instead of unilaterally determining course placement, the placement tests now become one of many diagnostic tools (along with high school coursework and individual student preferences) that advisors can use in helping students make the most well-informed decisions possible regarding their course schedules.

Although we have phrased our discussion largely in terms of passing rates, it is important to also consider the failure rates. That is, more than one-third of underprepared students are predicted to fail gateway English under the new legislation. Although students were given the responsibility of making their own enrollment choices, it is the colleges’ responsibility to provide these students with the resources and support to succeed. Because many underprepared students are now enrolling in gateway English, advisors and faculty should ensure that all students are referred to tutoring centers and additional support systems to help ensure their success. Integrating embedded tutors in classrooms and requiring attendance in writing labs or use of online tutoring may be one way to increase students’ awareness and use of available resources, particularly for students in gateway courses. Increased communication and coordination between gateway course faculty, DE faculty, and support personnel may also provide a bridge for students’ gaps in understanding between the different course levels (see Brower et al., 2017).

Because students at each level of preparation in reading and predicted are to pass at relatively similar rates as students in the same level of preparation in writing, it is possible that many of the skills students need to develop in reading and writing overlap. This may be a reason that some DE reforms have begun to combine developmental reading and writing courses into one course that covers both topics. For example, Edgecombe et al. (2014) found that students who enrolled in an accelerated reading and writing course had better short- and long-term outcomes than students in the two-course sequence of DE reading and writing. The state of Virginia has implemented two reading/writing developmental courses, a four-unit course for slightly underprepared students and an eight-unit course for more severely underprepared students, both just one semester long (Edgecombe, 2016). These findings may support the move toward additional models of combined reading and writing courses for underprepared students in Florida and across the country.

### Directions for future research

Estimating relationships between preparation levels and other measures of success are crucial to fully understanding how underprepared students are faring in this context of developmental reform. In addition to the course passing rates examined here, we are interested in how preparation is related to success in introductory courses throughout the social sciences and humanities majors and meta-majors. That is, how do underprepared students perform in their anthropology, psychology, sociology, language, and history courses when they enroll in DE or bypass it and enroll in the gateway course? How is gateway course success for underprepared students related to success in their major courses? Given that academic reading and writing are heavily integrated into most majors, additional attention paid to how these basic skills relate to success in major-course pathways is warranted.

Further, we call for an investigation of how underprepared students are succeeding when they have taken developmental courses, either in the same semester or prior to enrolling in the gateway course to compare success between students who did and did not enroll in a developmental course. Given the new instructional modalities of the developmental course offerings and research that touts their importance and effectiveness (Cho, Kopko, Jenkins, & Jaggars, 2012; Complete College America, 2016; Edgecombe, 2011; Hern, 2012; Jaggars, Hodara, Cho, & Xu, 2015; Okimoto & Heck, 2015; Sheldon & Durdella, 2009), the context of Florida’s DE reform warrants additional rigorous research of new instructional modalities and student success.

Whereas we recommend additional research to understand the full scope of Florida’s DE reform, this study provides evidence that underprepared students who choose to enroll in gateway courses can be successful. As more colleges and states begin to modify course placement, advising, and instructional practices, the findings presented here can provide useful information to policymakers, administrators, and instructors who work to improve student success.

## Disclosure of potential conflicts of interest

The opinions expressed are those of the authors and do not represent views of the Institute or the U.S. Department of Education, or the Gates Foundation. This manuscript has not been submitted elsewhere for publication. Submitted to the editor-in-chief for review May 11, 2017.

## Funding

The research reported here was supported by the Institute of Education Sciences, U.S. Department of Education, through Grant R305A160166 to Florida State University, and in part by a grant from the Bill & Melinda Gates Foundation.
